# The structural basis of hyperpromiscuity in a core combinatorial network of type II toxin–antitoxin and related phage defense systems

**DOI:** 10.1073/pnas.2305393120

**Published:** 2023-08-09

**Authors:** Karin Ernits, Chayan Kumar Saha, Tetiana Brodiazhenko, Bhanu Chouhan, Aditi Shenoy, Jessica A. Buttress, Julián J. Duque-Pedraza, Veda Bojar, Jose A. Nakamoto, Tatsuaki Kurata, Artyom A. Egorov, Lena Shyrokova, Marcus J. O. Johansson, Toomas Mets, Aytan Rustamova, Jelisaveta Džigurski, Tanel Tenson, Abel Garcia-Pino, Henrik Strahl, Arne Elofsson, Vasili Hauryliuk, Gemma C. Atkinson

**Affiliations:** ^a^Department of Experimental Medicine, Lund University, Lund 221 84, Sweden; ^b^Institute of Technology, University of Tartu, Tartu 50411, Estonia; ^c^Department of Molecular Biology, Umeå University, Umeå 901 87, Sweden; ^d^Department of Biochemistry and Biophysics and Science for Life Laboratory, Stockholm University, Solna 171 21, Sweden; ^e^Centre for Bacterial Cell Biology, Biosciences Institute, Newcastle University, Newcastle upon Tyne NE2 4AX, United Kingdom; ^f^Cellular and Molecular Microbiology, Faculté des Sciences, Université libre de Bruxelles, Brussels 1050, Belgium; ^g^Science for Life Laboratory, Lund 221 84, Sweden; ^h^Lund University Virus Centre, Lund 221 84, Sweden

**Keywords:** toxin, antitoxin, AlphaFold, phage, Panacea

## Abstract

Toxin-antitoxin (TA) systems are a large group of small genetic modules found in prokaryotes and their mobile genetic elements. Type II TAs are encoded as bicistronic (two-gene) operons that encode two proteins: a toxin and a neutralizing antitoxin. Using our tool NetFlax (standing for Network-FlaGs for toxins and antitoxins), we have performed a large-scale bioinformatic analysis of proteinaceous TAs, revealing interconnected clusters constituting a core network of TA-like gene pairs. To understand the structural basis of toxin neutralization by antitoxins, we have predicted the structures of 3,419 complexes with AlphaFold2. Together with mutagenesis and functional assays, our structural predictions provide insights into the neutralizing mechanism of the hyperpromiscuous Panacea antitoxin domain. In antitoxins composed of standalone Panacea, the domain mediates direct toxin neutralization, while in multidomain antitoxins the neutralization is mediated by other domains, such as PAD1, Phd-C, and ZFD. We hypothesize that Panacea acts as a sensor that regulates TA activation. We have experimentally validated 16 NetFlax TA systems and used domain annotations and metabolic labeling assays to predict their potential mechanisms of toxicity (such as membrane disruption, and inhibition of cell division or protein synthesis) as well as biological functions (such as antiphage defense). We have validated the antiphage activity of a RosmerTA system encoded by *Gordonia* phage Kita, and used fluorescence microscopy to confirm its predicted membrane-depolarizing activity. The interactive version of the NetFlax TA network that includes structural predictions can be accessed at http://netflax.webflags.se/.

## Significance

Toxin–antitoxin systems are enigmatic components of microbial genomes, with their biological functions being a conundrum of debate for decades. Increasingly, TAs are being found to have a role in defense against bacteriophages. By mapping and experimentally validating a core combinatorial network of TA systems and high-throughput prediction of structural interfaces, we uncover the evolutionary scale of TA partner swapping and identify toxic effectors. We validate the predicted toxin:antitoxin complex interfaces of four TA systems, uncovering the evolutionary malleable mechanism of toxin neutralization by Panacea-containing PanA antitoxins. We find TAs are evolutionarily related to several other phage defense systems, cementing their role as important molecular components of the arsenal of microbial warfare.

Toxin-antitoxin (TA) systems typically consist of two adjacent, often overlapping genes that encode a toxin whose expression causes growth arrest and a cognate antitoxin that negates the toxic effect (1). Based on the nature and mode of action of the antitoxin, TA systems are classified into eight types depending on the mechanism of toxin neutralization and whether the components are RNA- or protein-based (2). The most common group of proteinaceous TA pairs is type II, where the protein antitoxin directly binds to the protein toxin to sequester it into an inert complex (2).

The first TA operon to be discovered, *ccdAB*, was identified due to its stabilizing effect on plasmids (3). The rate of TA discovery has dramatically increased as high-throughput approaches for TA identification have been developed. Systematic experimental discovery of TAs was first achieved using shotgun cloning for identification of toxic ORFs (4). As the number of sequenced genomes and known TAs have grown, sensitive sequence searching and “guilt by association”—i.e., conserved colocalization of toxin and antitoxin as a bicistronic operon—have been used for discovery of new TA systems (5–12). Our bioinformatics-driven TA identification relies on analysis of gene neighborhood conservation using an approach that is sensitive enough to find remote similarity even in small, divergent proteins (9, 13, 14).

TA systems are ubiquitous in microbial life, and their wide distribution and extreme diversity of TAs has driven the search to discover the biological roles of these systems (2). Increasingly, TAs are being discovered to mediate defense against phages (15, 16), and large-scale exploratory and focused mechanistic approaches have rapidly advanced the field (16–22).

Being frequently horizontally transferred components of accessory genomes, TA systems have patchy distributions across genomes (5). It has long been known that type II toxins and antitoxins have a degree of modularity, in that they can swap partners through evolution (5, 8, 11, 24, 25). The hyperpromiscuous antitoxin domain Panacea is a striking example of how extensive TA partner swapping can be, with Panacea-containing antitoxins (PanA) being paired with dozens of different evolutionary and structurally unrelated toxin domains (PanTs) (14). This suggested that the Panacea domain may have inherent properties that enable it to neutralize multiple unrelated toxins through an unknown mechanism (14). However, a structural understanding of PanA-mediated neutralization has been lacking. Furthermore, while Panacea’s hyperpromiscuity is remarkable, it is unclear just how much this is paralleled in other antitoxins.

In this study, we have systematically explored the TA partner swapping network using NetFlax (standing for Network-FlaGs for toxins and antitoxins), an iterative implementation of our gene neighborhood analysis tool FlaGs (13), followed by experimental validation and characterization of TA systems. We have identified 3,597 systems within which there are 278 distinct homologous clusters of proteins in 275 distinct combinations of two-gene modules. We have structurally annotated our network of TA-like two-gene architectures through high-throughput prediction of TA complex structures using AlphaFold2 (26) implemented in the FoldDock pipeline (27). Focusing on the Panacea node of the network, we have validated our structural predictions through mutagenesis. We establish that Panacea is an evolutionally malleable domain that can both inhibit toxins through direct interaction and as serve as a platform for toxin neutralization by Panacea-associated ZBD (Zn^2+^-binding domain) and PAD1 (Panacea-Associated Domain 1) domains. The combinatorial network reveals close evolutionary relationships between classical type II TA systems and antiphage systems, specifically those that include the AAA ATPase and OLD_TOPRIM endonuclease domains such as those seen in PARIS (19), AbiLi (28) the Septu system (29), and ImmA protease-containing systems as seen in RosmerTA systems (30). We explore the network experimentally through validating 16 systems in toxicity neutralization assays and predict their potential mechanisms of toxicity through functional domain annotations and metabolic labeling assays. Finally, we validate the antiphage activity of a RosmerTA system encoded by *Gordonia* phage Kita, and use fluorescence microscopy to confirm its predicted membrane-depolarizing activity.

## Results

### The NetFlax Algorithm Reveals a Core Proteinaceous TA Network.

To uncover a core framework of the network of TA pairs, we developed the computational tool NetFlax that identifies TA-like gene architectures in an unsupervised manner and generates a TA domain interaction network. The NetFlax principle is that if one partner gene of a TA system is found in a conserved two-gene neighborhood with an alternate partner, this is predicted as a new pair, and after “hopping” to this new partner, more partners can be found in the same way (*SI Appendix*, *SI Text* and Fig. S1). We set a requirement that each new pair must be conserved in at least eight representative genomes to be allowed to hop to a new node. As this stringency leads to missing some less well conserved systems, we improved sensitivity through adding a final guilt by association hop for each node, which only required a system to be conserved in two representative genomes.

**Fig. 1.**
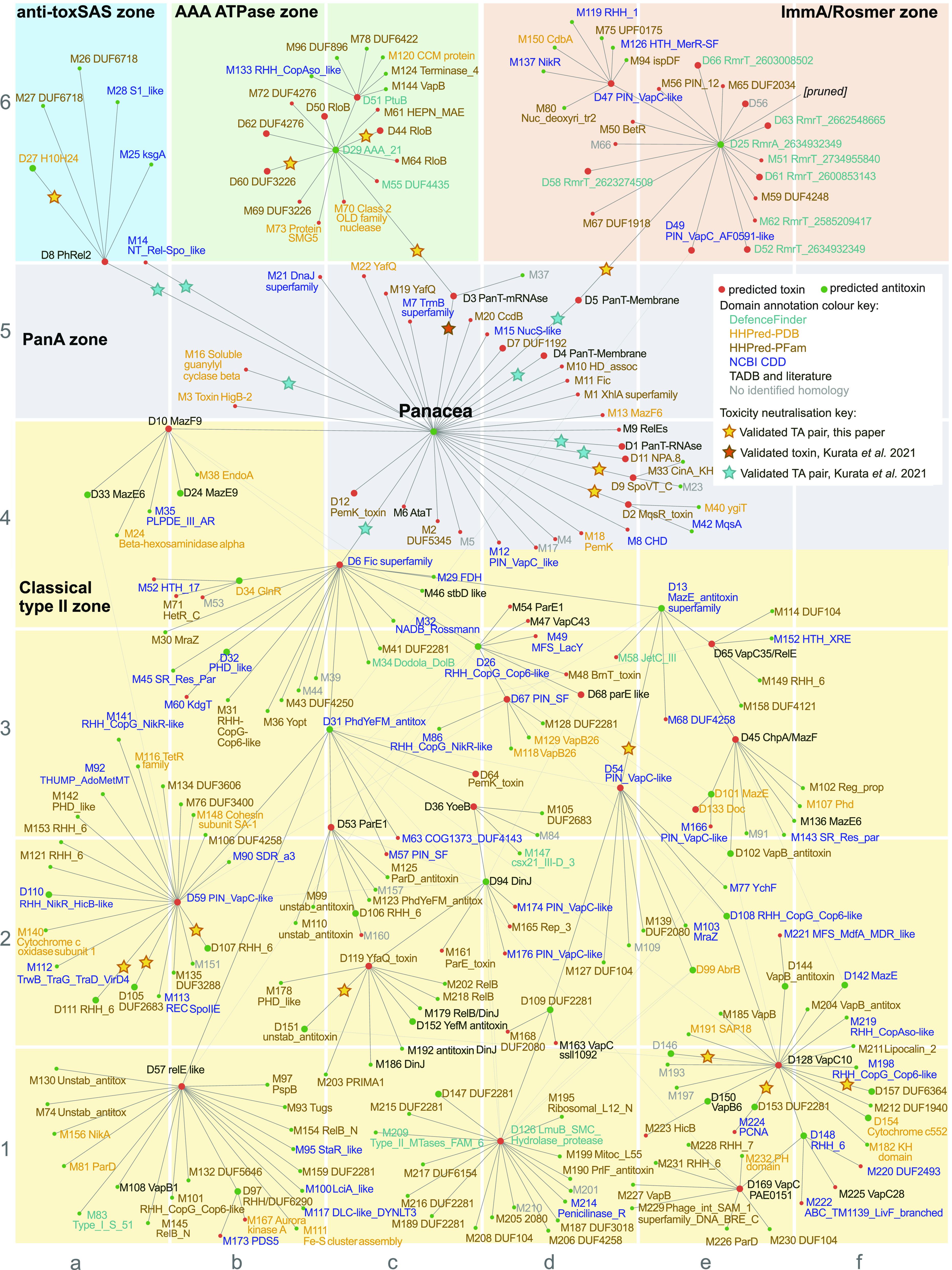
The core proteinaceous TA network. The network shows connections of NetFlax-predicted toxin and antitoxin-like protein clusters across microbial life. The starting input was the Panacea domain. Green circles are predicted antitoxins and red circles are predicted toxins. Yellow stars show toxins and antitoxins validated here (Dataset S1). Dark orange and cyan stars show, respectively, toxins and TAs that have been previously validated in Kurata *et al*. (14). Predicted toxin and antitoxin domains are annotated based on sequence homology searches (see Materials and Methods for details and references).

NetFlax finished hopping after eight hopping steps, converging on a final network, having reached dead-ends for all the network lineages (Fig. 1). We initially identified 79 clusters conserved in a minimum of eight genomes. These we call D nodes (standing for central Domain nodes). After the subsequent less strict node analysis allowing conservation in two genomes with no onward hopping, we identified 234 additional nodes, which we refer to as M nodes, for Mininodes. In total, we identified 314 nodes. Toxin/antitoxin assignments are made by virtue of their lineages from the original Panacea antitoxin domain, assuming that the hopping goes from antitoxin to toxin to antitoxin etc. This assumption seems to work well on the whole; in the classical type II part of the network, our annotation of whether the cluster is a toxin or antitoxin domain matches that in the TADB database (31), and domain annotations (Dataset S1). However, we cannot be sure that our annotations hold true for the termini of the network. One extended lineage of three D nodes leading from the Rosmer/ImmA zone (D41, D95, D127, and D132 associated with 32 combined M nodes) became particularly complicated with node domain fusions, making our ability to predict toxins and antitoxins troublesome. Therefore, we decided on balance to “prune” this lineage from the core TA network of Fig. 1 (however, these lineages and their data are still available in the unpruned interactive network http://netflaxunpruned.webflags.se/ and Dataset S1).

**Fig. 2.**
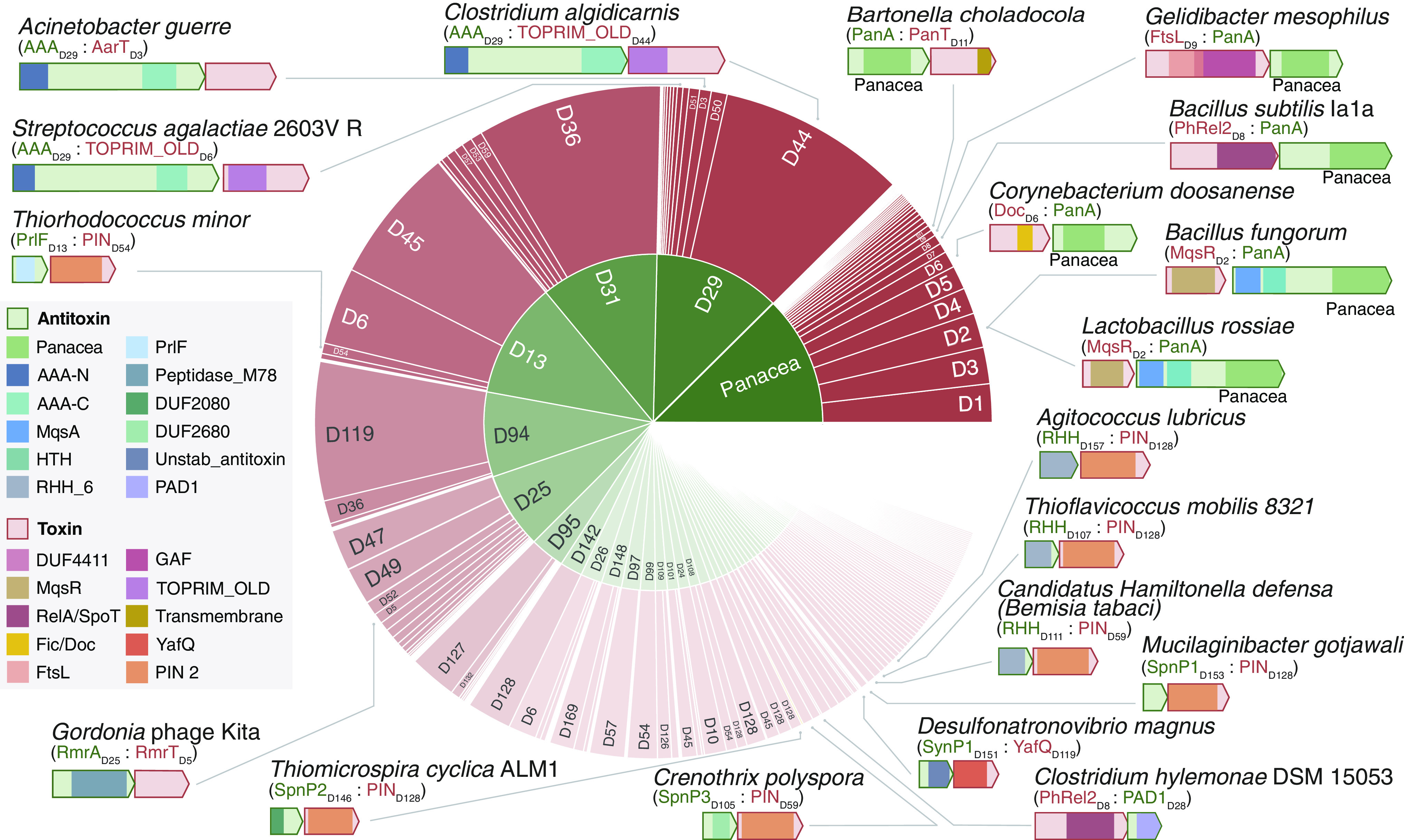
The diversity of systems in the core NetFlax TA network. The size of the sections represents the number of proteins in each cluster (node) of Fig. 1. The inner ring shows antitoxin nodes, while the outer ring shows associated toxin nodes. The block arrows represent open reading frames, drawn to scale. Colored boxes within the arrows indicate domains, colored according to the legend on the left.

Our final core TA network (Fig. 1 and http://netflax.webflags.se/) represents the most conserved systems of the 24,474 representative predicted proteomes. The network comprises 278 nodes, of which 107 are predicted to be toxins, and 171 are predicted to be antitoxins. These fall into 275 distinct TA node combinations. It is useful to roughly divide the network into five topological zones: i) the Panacea domain–containing systems at the core of the network, including six systems experimentally validated in our previous analyses (Dataset S1) (14), ii) the anti-toxSAS zone containing toxins related to RelA/SpoT alarmone synthetases that likely modify tRNA (nodes D8 and M14), plus their antitoxins, iii) a zone containing a hub AAA ATPase antitoxin domain (D29, Fig. 1 coordinates c6), iv) a zone containing a hub ImmA protease antitoxin domain (D25, Fig. 1 coordinates e6) and v) the largest zone of the network where many classical type II TA systems are found, with many interconnections among nodes indicating considerable partner swapping. Panacea-containing systems are the largest group of our TAs (Fig. 2), closely followed by D29 (AAA ATPase-like) and D31 (Phd-related antitoxins). Most node pairs favor a particular gene order of either toxin first or antitoxin first (the latter being the most common overall, Dataset S1). D25 (ImmA/Rosmer) and Panacea antitoxin nodes are unusual in their gene order variability (58% and 78% toxin first, respectively).

### Phage Defense Systems Are Widespread in the NetFlax TA Network.

For each node in the NetFlax network, we made functional predictions for a protein representative by searching with domain models including those from DefenceFinder, a database of phage defense systems (25). The latter search revealed that the AAA ATPase and ImmA zones are particularly enriched in phage defense systems. The AAA ATPase domain of node D29 (Fig. 1 coordinates c6) is found in a number of defense systems, such as AriA of the PARIS system (19), GajA of the Gabija system (32) and PtuA of the Septu system (29). GajA is a sequence-specific ATP-dependent DNA endonuclease that is inhibited by dNTP and NTP nucleotides (14). It is a two-domain protein, with an N-terminal AAA domain and a C-terminal TOPRIM (topoisomerase-primase) endonuclease domain, closely related to OLD (Overcome Lysogenization Defect) families (14). In our network, we see the latter domain can be associated with the AAA domain as a separate protein (node M70, coordinates c6). This is the same two-gene architecture as seen in AriAB of the PARIS system (19). Among the other nodes linked to D29 is D44, homologous to RloB. The RloB protein family has been observed in type I restriction-modification operons (33), and the AbiLii protein, which is part of a plasmid-encoded phage abortive infection mechanism (28). HHPred (34) indicates the RloB domain is also related to OLD_TOPRIM domains. The node D51 is homologous to the HNH nuclease domain, as seen in Septu protein PtuB (Dataset S1). Thus D29 and its cognate D51 together constitutes a similar two-domain PtuAB Septu system architecture previously identified in *Bacillus thuringiensis* (29). The presence of the HEPN nuclease domain in node M61 indicates a general tendency for nuclease domains to be associated with AAA ATPase domains.

The ImmA protease domain which NetFlax predicts as an antitoxin (D25, Fig. 1 coordinates e6) was recently confirmed as such in the diverse phage defense RosmerTA systems, where different toxins (RmrTs) are paired with the protease domain–containing antitoxin (RmrA) (18, 30, 35) (Fig. 1). The ImmA domain is named after the protein encoded on a conjugative transposon of *Bacillus* *subtilis* (36). ImmA is an antirepressor that cleaves an HTH (Helix-Turn-Helix) domain–containing repressor (ImmR) to allow the expression of an integrase (36). Indeed, proteins within the D25 node often possess a small N-terminal HTH domain in addition to the protease domain, suggesting a similar HTH cleavage mechanism of regulation in Rosmer-like TAs. Again, we see the involvement of nucleases in our predicted systems, this time in an association of D25 with PIN-like domains, the most diverse and ubiquitous nuclease superfamily, often seen as toxin components of TAs (37, 38).

Phage defense domains appear in other parts of the NetFlax network. In addition to the AAA and ImmA/Rosmer zones that are clearly phage defense related, we have recently found that toxSASs can protect against phages (22). Additionally, the identification of DefenceFinder domains in the Classical type II zone, along with evidence for classical TA domains in phage defense (39, 40) shows how intricately TAs in general are associated with phage defense.

### NetFlax TAs Are Found across the Prokaryotic Tree of Life, and in Tailed Bacteriophages.

NetFlax-predicted TAs are found in all major phyla of bacteria and archaea (*SI Appendix*, Fig. S2 and Dataset S1). Most NetFlax TAs were found in Pseudomonadota (formally known as Proteobacteria)—particularly Gammaproteobacteria, reflecting the bias of RefSeq toward these taxa. The pseudomonad *Thiobaca* *trueperi* has the most NetFlax-predicted TAs (eight). NetFlax TAs are also found across the archaeal tree of life, with representatives in the phyla Euryarchaeaota, Crenarchaeota, Thaumarchaeota, Candidatus Thermoplasmota, and Candidatus Koracheota. Within viruses, TAs were only predicted in Uroviricota (tailed bacteriophages). Our identification of 13 NetFlax TAs in phages (Dataset S1) is likely a significant underestimate as bacteria-encoded systems can be resident on prophages integrated into the bacterial chromosome, for example the toxSAS CapRel (22).

### AlphaFold2 Confidently Predicts the Structure of Binary TA Complexes.

TAs are excellent targets for modern deep-learning structural prediction methods—not just of single proteins—but of complexes. This is because type II systems necessarily form tight complexes to keep the toxin in check, with a coevolutionary signal in the interface region (41). We have run AlphaFold2 on a high throughput basis with the FoldDock pipeline (27) to predict the structure of all 3,597 protein pairs (3,277 after pruning). To keep predictions computationally feasible, we predict binary TA dimers, not higher order oligomers, rationalizing that even in larger complexes, there must be an interface between the toxin and antitoxin. The reliability of the structures of the complexes is assessed using the pDockQ score, which takes into account the number of interface contacts, and the plDDT reliability scores from AlphaFold2 for those regions. All structures and their scores are available on the interactive network (http://netflax.webflags.se/). Recapitulation of TA folds previously solved with X-ray crystallography indicates these predictions are reliable (*SI Appendix*, Fig. S3). We determined the distribution of model confidence (pDockQ scores) of TA pairs compared to random pairs. The TA predictions are much better than random predictions for the same set and roughly 50 to 60% of the complexes are well modeled (*SI Appendix*, Fig. S4).

### Recurrent Structural Folds Appear across the NetFlax Network.

The NetFlax algorithm includes a cross-checking step to determine whether each potential cluster is unique or similar to any cluster identified during the previous hopping rounds (*SI Appendix*, Fig. S1*B*). Despite this, we found multiple clusters in the classical type II zone of the network with similar annotations, suggesting they may be homologous despite not clustering together. For example, the Phd, ParE, and PIN domain appear multiple times in the network (Fig. 1). Sequence alignments show that these unclustered but related nodes are clearly distinct in terms of sequence (including insertions and deletions, *SI Appendix*, Fig. S5), but that they are similar enough that they have the same three-dimensional fold. To systematically address this, we clustered all our predicted structures and annotated our network to show nodes that can be confidently classed as sharing a common fold (*SI Appendix*, Fig. S6). We find many of our predicted TAs can be clustered into 18 distinct folds, the most common being ATPase, MazF/PemK, PIN, RelE/ParE, Phd/YefM, and a common fold of Rosmer toxins (25). Structural alignments of the most common folds are shown in *SI Appendix*, Fig. S7. This supports previous observations that toxins and antitoxins that are diverse at the sequence level can have the same structural fold (42). A similar conservation of domains is also seen in phage defense systems (43).

### PanA-Containing TAs: The Roles of Individual Antitoxin Domains in Toxin Neutralization.

**Fig. 3.**
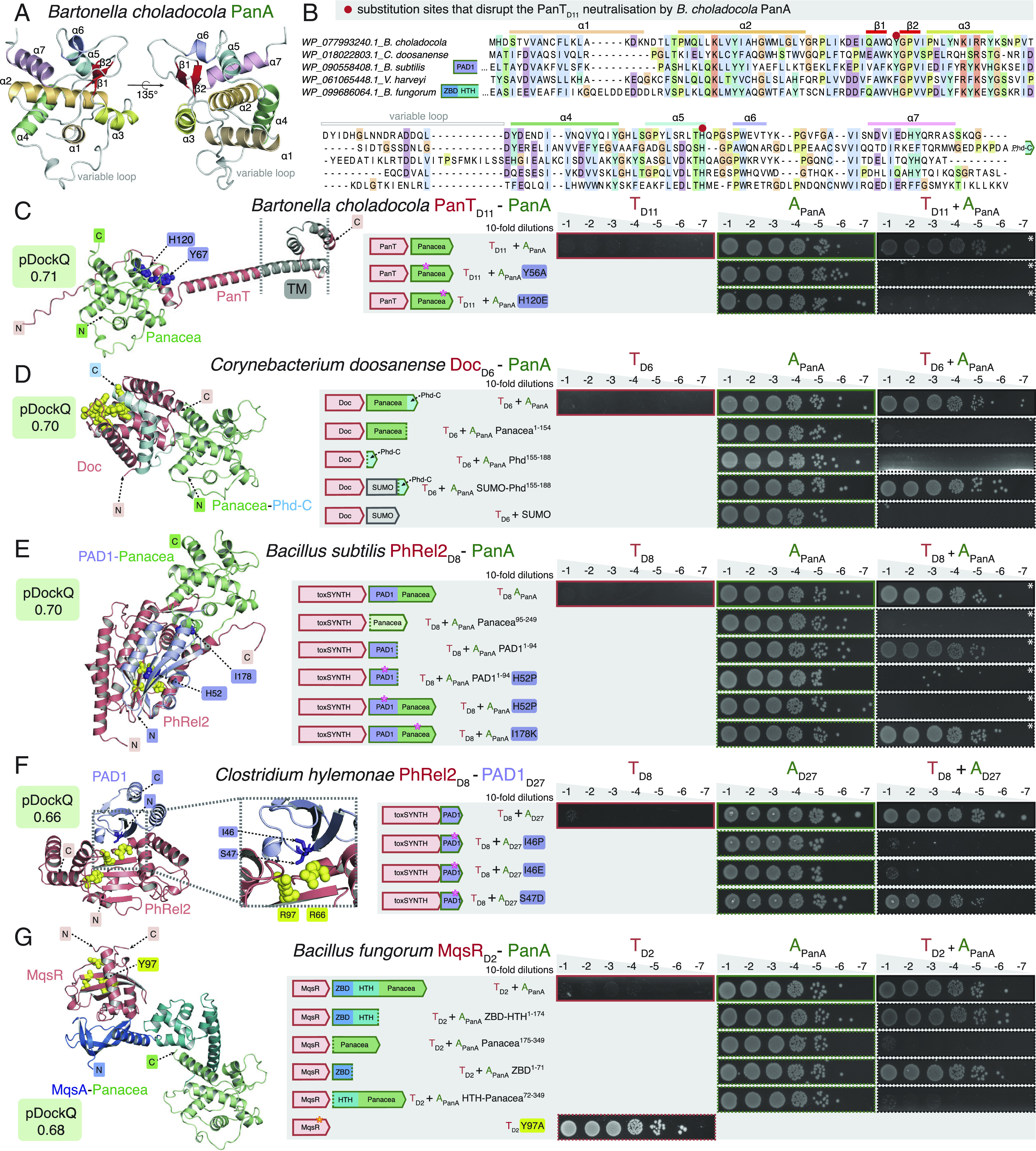
PanA antitoxins neutralize PanT toxins either via Panacea domain directly or via additional N-terminal domains: Phd-C, PAD1, and MqsA. (*A*) AlphaFold-generated structural model of *B. choladocola* PanA. (*B*) Alignment of Panacea domains from representative PanA antitoxins. (*C*–*G*) Mutational probing of AlphaFold-generated structural models. In toxicity neutralization assays overnight cultures of *E.* *coli* strains transformed with pBAD33 and pMG25 vectors or derivatives expressing putative *panT* toxins and *panA* antitoxins, correspondingly, were adjusted to OD_600_ 1.0, serially diluted from 10^1^- to 10^8^-fold and spotted on LB medium supplemented with appropriate antibiotics and inducers [0.2% arabinose for toxin induction as well as either 50 or 500 µM (white asterisk) IPTG for antitoxin induction]. Predicted transmembrane domains (TM) are shown in gray, and the active site of the toxin is highlighted in yellow. Introduced substitutions in the antitoxin are shown in purple. Protein accessions are in *SI Appendix*, Table S1.

We focused on three previously experimentally validated PanAT TAs: *Bartonella choladocola* (previously *Bartonella apis*) PanT_D11_:PanA, *Corynebacterium doosanense* Doc_D6_:PanA, *B.* *subtilis* Ia1a toxSAS PhRel2_D8_:PanA as well as one previously unexplored PanAT, *Bacillus fungorum* MqsR_D2_:PanA. The subscript D number refers to the node in Fig. 1. All NCBI protein accession numbers of TAs characterized in this paper are shown in *SI Appendix*, Table S1. In all of the PanAT systems, the Panacea domain is predicted to have the same compact architecture comprised of α-helices α1-α7 and β-strands β1 and β2 (Fig. 3 *A* and *B*). Despite these PanATs having dramatically different toxins, these four TA structures are predicted with confidence (pDockQ scores from 0.68 to 0.71). As selected PanAT systems differ in their antitoxin architecture (Fig. 3 *C*–*E* and *G* and see below), mutational analysis of the set allows us to interrogate the function of the Panacea domain in PanAs: Does it mediate toxin neutralization directly or is this achieved by additional domains?

The structure of *B. choladocola* PanT_D11_:PanA suggests that Panacea can, indeed, directly neutralize the toxin (Fig. 3*C*). In this case, Panacea is predicted to form a contact with the N-terminal unstructured region as well as a short α-helix that precedes the PanT_D11_ predicted transmembrane region (14). Substitutions Y56A (N-terminally adjacent to β2) and H120E (C-terminal end of α5) that were designed to disrupt this interface do, indeed, render *B. choladocola* PanA unable to neutralize the toxin, thus supporting the structural model. Both of these substituted residues are located in the conserved structural core of the Panacea domain (Fig. 3*B*).

In the case of *C. doosanense* PanAT, the two additional C-terminal helices decorating the Panacea core of the PanA antitoxin are predicted to make extensive contacts with the Doc_D6_ toxin (Fig. 3*D*). The globular Panacea domain itself is not predicted to be involved in neutralization. These two C-terminal helices are structurally analogous to those found in the C-terminal extension of the *E. coli* Phd antitoxin that inhibits the Doc toxin (44). Therefore, we refer to this element of *C. doosanense* PanA as the Phd-C domain. *E. coli* Doc is a kinase that phosphorylates EF-Tu to abrogate cellular protein synthesis (45); *C. doosanense* Doc_D6_ similarly targets translation (14), and the active site residues are conserved among the two proteins (Fig. 3*D*). The Phd-C domain of PanA directly interacts with the active site of the toxin. Truncation of the Phd-C domain renders *C. doosanense* PanA unable to neutralize the toxin (Fig. 3*D*). Expression of the isolated Phd-C domain does not neutralize the toxin, which could be due to the intrinsic instability of the element. To test this hypothesis, we fused Phd-C with a stabilizing N-terminal SUMO tag, and as predicted, the resulting construct can readily neutralize Doc_D6_, despite lacking the Panacea domain. No neutralization was observed in the control experiment with SUMO alone. Collectively, these results suggest that Panacea can serve as an accessory domain, with neutralization being mediated by a dedicated separate domain.

Next, we characterized the *B.* *subtilis* Ia1a PhRel2_D8_:PanA system. In our previous analysis of the Panacea domain distribution, we identified a domain that we named the PAD1 domain, standing for Panacea-associated domain 1 (14). Apart from two strains of Ruminococcaceae where the putative toxin is an ATPase, PAD1-Panacea multidomain PanA antitoxins are only found paired with toxSASs such as PhRel2_D8_, where it is the most widespread antitoxin for this kind of toxin in the NetFlax network. The second most widespread is NetFlax domain D27 (Fig. 1). Remarkably, structural alignment of the toxSAS:D27 of *Clostridium* *hylemonae* DSM 15053 with toxSAS:PAD1-PanA of *B.* *subtilis* Ia1a, fused TA CapRel (22) showed that D27, PAD1, and pseudo-ZBD are predicted to have the same fold, and share the same interface with the toxSAS toxin (*SI Appendix*, Fig. S8). Importantly, it is PAD1 that forms most of the contacts with the PhRel2_D8_ toxin (Fig. 3*E*). Strikingly, *B.* *subtilis* Ia1a PAD1 domain alone—with Panacea removed—can neutralize the toxin, thus directly supporting the structural prediction. Furthermore, H52P substitutions that are predicted to break the PAD1:PhRel2_D8_ interface completely abrogate the neutralization, both in the context of full-length PanA and isolated PAD1. Finally, the I178K substitution located on the Panacea: PhRel2_D8_ interface did not affect the efficiency of neutralization. To further support the role of PAD1 as a dedicated toxin-neutralizing domain, we have, via toxicity neutralization assays, validated the *C. hylemonae* DSM 15053 TA system comprised of PhRel2_D8_ and the PAD1_D27_ antitoxin (Fig. 3*F*). As the *C. hylemonae* antitoxin naturally lacks the Panacea domain, this observation further supports PAD1 being a directly neutralizing antitoxin element. Substitutions predicted to compromise the PAD1:PhRel2_D8_ interface either weakened (S47D) or completely abrogated the neutralization (I46P and I46E).

Finally, we have dissected the *B. fungorum* MqsR_D2_:PanA system (Fig. 3*G*). MqsR is an RNase (42, 46) that is neutralized by antitoxin MqsA comprised of an N-terminal Zn^2+^-binding domain (ZBD) and C-terminal HTH (42). Substitution of the predicted catalytic Y97 residue of *B. fungorum* MqsR_D2_ renders it nontoxic, supporting the functional annotation. While the ZBD interacts with MqsR and inhibits it without directly interacting with the RNase active site, the HTH region dimerizes and acts as a transcriptional autoregulator of the *mqsRA* operon. *B. fungorum* PanA also contains the ZBD-HTH domain composition characteristic of the MqsA antitoxin, with the Panacea domain added to the C terminus. Our truncation analysis shows that, indeed, also in the case of *B. fungorum* PanAT, the ZBD directly mediates neutralization of MqsR_D2_. While both isolated ZBD and ZBD-HTH segments efficiently neutralize MqsR_D2_, neither Panacea alone nor HTH-Panacea are sufficient for neutralization. We used AlphaFold2 to predict homodimerization of our validated antitoxins (*SI Appendix*, Fig. S9). We see good support for HTH-mediated homodimerization of *B. fungorum* PanA. Note that in the HTH-containing PanA from *Lactobacillus rossiae* the Panacea domain also appears to be involved in homodimerization (*SI Appendix*, Fig. S9*A*).

Collectively, our results demonstrate that while Panacea can act as a direct toxin neutralizer, it is unlikely to act as such in PanA antitoxins that contain additional dedicated neutralization domains such as PAD1 or ZBD. Furthermore, the example of *B. fungorum* MqsR_D2_:PanA system suggests that Panacea probably does not act as a transcriptional autoregulator of *panAT* operons either, as *B. fungorum* PanA contains a dedicated DNA-binding regulatory domain, HTH (as do many other PanAs, ref. 14). Therefore, we favor the hypothesis that the Panacea domain acts as a sensor responding to—as yet unknown—TA-activating cues.

### Experimental Exploration of the NetFlax Network.

We have validated 16 TA pairs in toxicity neutralization assays (Fig. 1). For 13 of them, we performed metabolic labeling assays with ^35^S methionine (a proxy for inhibition of translation), or ^3^H uridine (a proxy for inhibition of transcription), or ^3^H thymidine (a proxy for inhibition for replication). Ten additional systems did not show any toxicity in *E. coli* and were not pursued further (*SI Appendix*, Fig. S10).

**Fig. 4.**
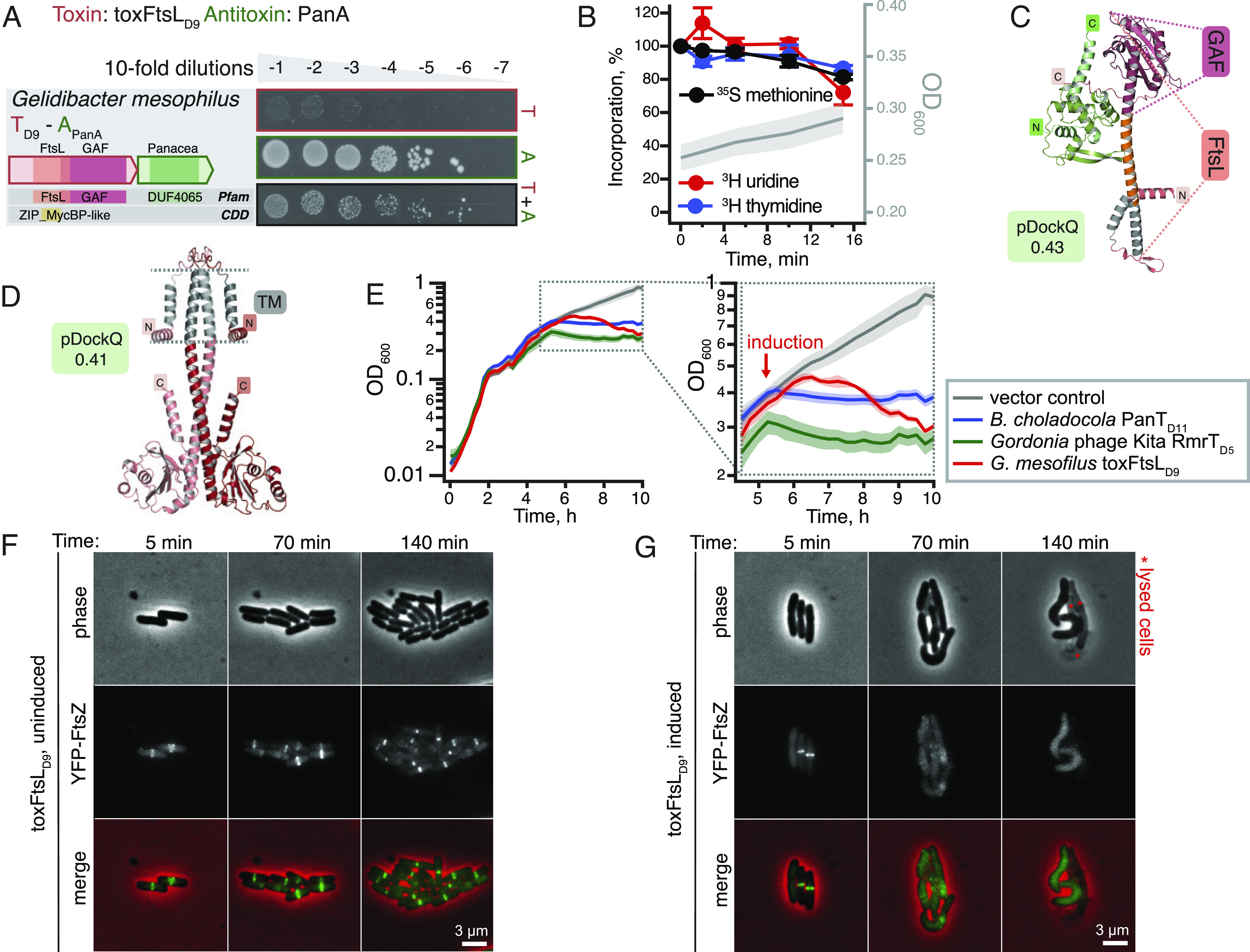
*G.* *mesophilus* toxFtsL_D9_ is a slow-acting PanT toxin with partial homology with the FtsL component of bacterial divisome. (*A*) Validation of the *G.* *mesophilus* toxFtsL_D9_:PanA TA through toxicity neutralization assay. (*B*) Metabolic labeling assays with wild-type *E.* *coli* BW25113 expressing *G.* *mesophilus* toxFtsL_D9_. (*C*) PanAT structural prediction of *G.* *mesophilus* toxFtsL_D9_:PanA TA pair. toxFtsL_D9_ is predicted to have two partially overlapping domains: The α-helical FtsL-like region is highlighted with orange dotted guide lines, and the GAF domain is indicated with dark pink dotted guide lines. (*D*) Structural prediction of the toxFtsL_D9_ dimer. Predicted transmembrane (TM) helical regions are shown in gray on *C* and *D*. (*E*) Delayed growth inhibition and cell lysis by *G. mesophilus* toxFtsL_D9_. Growth assays of *E. coli* BW25113 cells expressing *G. mesophilus* toxFtsL_D9_, *B. choladocola* PanT_D11_ or *Gordonia* phage Kita RmrT_D5_ as well as a vector control strain harboring pBAD33 and pMG25 in MOPS liquid medium supplemented with 0.5% glycerol and 25 µg/mL each 20 amino acids. Expression of toxins was induced with 0.2% arabinose at OD_600_ of around 0.4. (*F* and *G*) Fluorescence and phase contrast time lapse microscopy of *E. coli* BW25113 cells expressing YFP-FtsZ in the absence (uninduced, *E*) and presence (induced with 0.2% arabinose, *F*) of *G*. *mesophilus* toxFtsL_D9_. Note the toxFtsL_D9_-induced delocalization of the main divisome scaffold protein FtsZ, and the associated cell elongation. For the complete time lapse data, see Movie S1. For quantification of cell elongation and additional controls, see *SI Appendix*, Figs. S11 and S12.

The TA system from *Gelidibacter mesophilus* is comprised of a relatively large (281 aa) toxin T_D9_ (toxFtsL_D9_) paired with a PanA antitoxin (Fig. 4*A*). Similarly to *B. choladocola* PanT_D11_:PanA, *G. mesophilus* PanA is comprised of a stand-alone Panacea domain that directly neutralizes the toxin. While the toxin is clearly very efficient in abrogating the formation of bacterial colonies on solid LB plates, induction in liquid culture does not result in rapid growth inhibition nor do we see any dramatic effects in metabolic labeling assays (Fig. 4*B*). The toxin contains a GAF (cGMP-specific phosphodiesterases, adenylyl cyclases, and FhlA) domain and an α-helical FtsL-like domain, which is predicted to dimerize and be localized to the cell membrane (Fig. 4 *C* and *D*). FtsL is an essential component of bacterial divisome, which forms a trimeric complex with FtsB and FtsQ via leucine zipper-like motifs (47). Given the partial homology with FtsL, we propose naming the *G. mesophilus* T_D9_ toxin toxFtsL_D9_. It is tempting to speculate that *G. mesophilus* T_D9_ could act by directly interfering with the cell division process. Experiments with liquid cultures of *E. coli* expressing *G. mesophilus* toxFtsL_D9_ lend support to this hypothesis: After an hour of uninhibited growth, the OD_600_ increase stops and then the culture collapses, suggestive of cell lysis (Fig. 4*E*). Microscopy experiments show that expression of toxFtsL_D9_ indeed results in cell filamentation and inhibition of divisome assembly (Fig. 4 *F* and *G*, *SI Appendix*, Fig. S11, and Movie S1). Crucially, this is not caused by partial membrane depolarization, which can interfere with the bacterial cell division process (*SI Appendix*, Fig. S12) (48). Inhibition of cell division is an established mode of action for TA toxins with *E. coli* toxin CbtA directly targeting FtsZ and MreB (49).

**Fig. 5.**
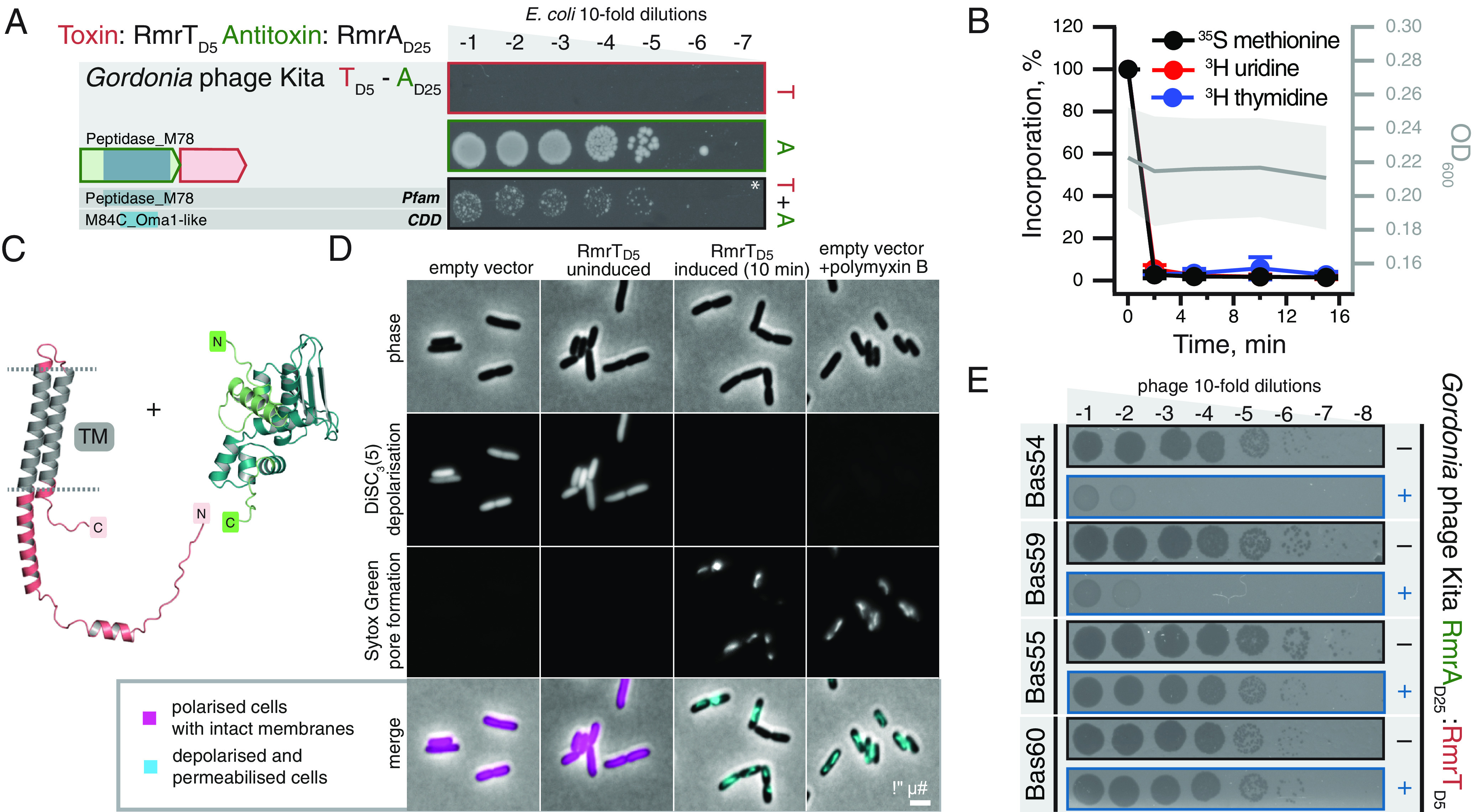
The RosmerTA antiphage defense system from *Gordonia* phage Kita. Domain organization and TA validation through toxicity neutralization assays (*A*), metabolic labeling assays with toxins expressed in wild-type *E.* *coli* BW25113 (*B*) and AlphaFold-generated structural models (*C*) for RmrT_D5_ and RmrA_D25_ TA from *Gordonia* phage Kita. Predicted TM regions of the toxin are shown in grey. Liquid culture experiments with RmrT_D5_ are shown on Fig. 4*E*. (*D*) Phase contrast and fluorescence images of *E.* *coli* BW25113 cells colabeled with the membrane potential-sensitive dye DISC_3_(5) and the membrane permeability indicator Sytox Green. Note the depolarization and pore formation induced by RmrT_D5_ that is comparable to the pore forming antibiotic Polymyxin B. For quantification of membrane depolarization and pore formation triggered by RmrT_D5_, see *SI Appendix*, Fig. S13. (*E*) *E.* *coli* BW25113 transformed with either empty pBR322 derivative, pJD1423 (VHp1423), (−) or pJD1423 derivative expressing *Gordonia* phage Kita RosmerTA from P_tet_ promoter (+) was challenged with ten-fold serial dilutions of BASEL coliphages (50). The full screen is shown on *SI Appendix*, Fig. S14, from which here we show results for Bas54 and Bas60 (defense) as well as Bas55 and Bas59 (lack of defense).

The TA system from *Gordonia* phage Kita is a new member of the RosmerTA family (18, 30, 35) (Fig. 5*A*). The RmrA _D25_ protease antitoxin is paired with a D5 toxin, which has no detectable similarity to other protein families. Metabolic labeling assays show rapid and dramatic abrogation of translation, transcription, and replication upon expression of the Kita phage D5 toxin (Fig. 5*B*). The toxin is not fully neutralized by the antitoxin, and the structure of the TA complex cannot be reliably predicted by AlphaFold (pDockQ score of 0.05) (Fig. 5*C*). The C-terminal region of the toxin is predicted to be localized to the cellular membrane (Fig. 5*C*). In liquid culture experiments, expression of RmrT_D5_ immediately inhibits bacterial growth without causing a consequent collapse of OD_600_; the toxin is likely to share the mechanism of toxicity with membrane-depolarizing *B. choladocola* PanT_D11_ (14) that we used as a control (Fig. 4*E*). Indeed, the expression of RmrT_D5_ results in rapid membrane depolarization and increased permeability indicating membrane pore formation (Fig. 5*D* and *SI Appendix*, Fig. S13). Following the nomenclature for RosmerTA toxins (18, 30, 35), we renamed the Kita phage toxin RmrT_D5_. As other RosmerTA systems have been shown to be phage defense systems (18, 30, 35), it is likely that the Kita phage RmrTA has a similar function. To test this, we carried out phage infection assays using the BASEL coliphage collection (50) and find that RmrTA provides potent but narrow spectrum defense against *Myoviridae*: it counters Bas54 and Bas59 but not to closely related Bas55 and Bas60 (Fig. 5*E*)—or any other BASEL coliphages (*SI Appendix*, Fig. S14). We further confirm protection against Bas59 in liquid culture infection assays (*SI Appendix*, Fig. S15).

**Fig. 6.**
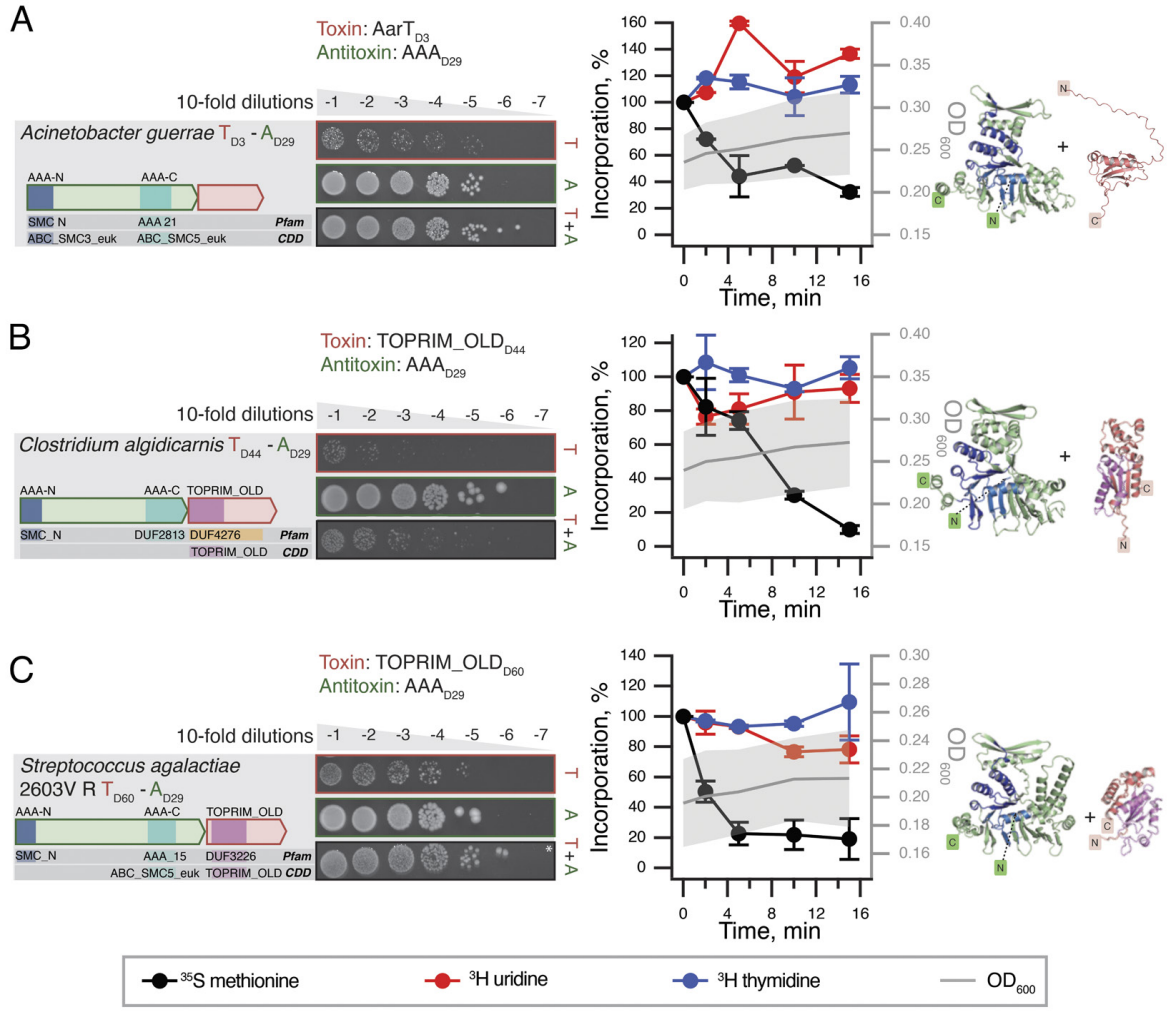
AAA-neutralizd putative Abi phage defense systems. Domain organization and TA validation through toxicity neutralization assays (*Left*), metabolic labeling assays with toxins expressed in wild-type *E.* *coli* BW25113 (*Center*) and AlphaFold-generated structural models (*Right*) for AAA_D29_-neutralized TAs: (*A*) *A.* *guerrae* AarT_D3_:AAA_D29_ (*B*) *C. algidicarnis* TOPRIM_OLD_D44_:AAA_D29_ and (*C*) *S. agalactiae* 2603V R TOPRIM_OLD_D60_:AAA_D29_.

The TA system from *Acinetobacter* *guerrae* is composed of a toxin T_D3_ that has no detectable hits with HHPred. However, it has the same fold as mRNA interferases (*SI Appendix*, Fig. S6), paired with an AAA ATPase A_D29_ antitoxin (Fig. 6*A*). We refer to the toxin as AarT for AAA-associated RNAse-like toxin. Metabolic labeling experiments suggest that the toxin targets protein synthesis as its expression inhibits ^35^S methionine incorporation with concurrent increase in ^3^H uridine, a pattern that is characteristic for translation-targeting toxins and antibiotics, and supporting an identity as an mRNAse or tRNase (14). Similar neutralization architecture was predicted for D_29_ AAA antitoxins from *Clostridium algidicarnis* (Fig. 6*B*) and *Streptococcus agalactiae* 2603V R (Fig. 6*C*). These two AAA antitoxins are paired with a TOPRIM_OLD domain. The *Lactococcus lactis* AbiL is a bicistronic plasmid-encoded phage system that acts thorough abortive infection elicited by the TOPRIM_OLD toxic effector AbiLii (28). We speculate that the three AAA-neutralized TA pairs are also Abi phage defense systems. AlphaFold2 modeling does not give a convincing interface for AAA antitoxins and their toxins (pDockQ score of 0.24 to 0.35), which may be because phage defense AAA-containing systems form large multimeric complexes, as is seen with AAA-containing RADAR (51, 52). Higher order complex formation of NetFlax-predicted AAA antitoxins is supported by our AlphaFold predictions of homodimerization (*SI Appendix*, Fig. S9*C*). A phage immunity screen of *E.* *coli* expressing *A.* *guerrae* AarT_D3_:AAA_D29_ versus the BASEL coliphage collection did not yield any hits. This does not rule out defense in the natural host; the mechanism of phage sensing and defense may be specific for *Acinetobacter* phages, or rely on host factors not present in *E.* *coli* (*SI Appendix*, Fig. S16).

**Fig. 7.**
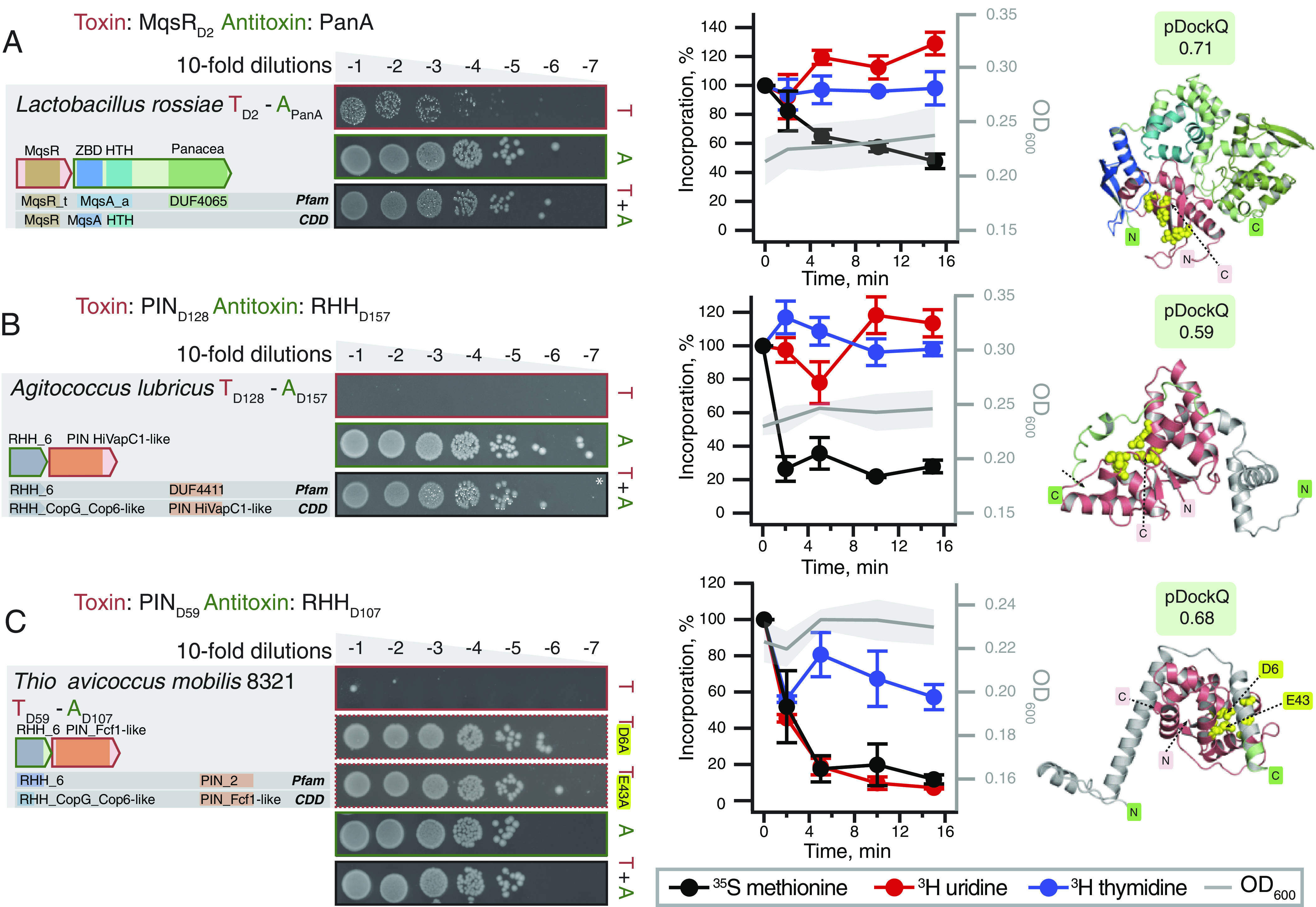
Representative NetFlax TA systems with nuclease effectors: MqsR_D2_, PIN_D128_ and PIN_D59_. Domain organization and TA validation through toxicity neutralization assays (*Left*), metabolic labeling assays with toxins expressed in wild-type *E.* *coli* BW25113 (*Center*) and AlphaFold-generated structural models (*Right*) for TAs with diverse nuclease effectors: (*A*) *L.* *rossiae* MqsR_D2_:PanA, (*B*) *A. lubricus* PIN_D128_:RHH_D157_ and (*C*) *T. mobilis* 8321 PIM_D59_: RHH_D107_. Active sites are highlighted with yellow spheres on the structures.

Finally, we have validated 10 TA pairs of nuclease toxins (MqsR_D2_, PIN/VapC-like and YafQ_D119_) paired with diverse antitoxins (PanA, PerlF_D13_, SpnP3/DUF2680_D105_, RHH_6_D107_, RHH_6_D111_, SpnP2/DUF2080_D146_, SynP1_D151_ and RHH_6_D157_), for which AF2 structural models suggest multiple mechanisms of direct and indirect toxin neutralization (Fig. 7 and *SI Appendix*, Figs. S17 and S18). While the structures were predicted as binary complexes, RHH (Ribbon-Helix-Helix) domain is a well-characterized dimeric DNA-binding transcriptional regulator employed by numerous antitoxins such as CcdA (53) and FitA (54). Dimers of RHH-containing antitoxins can be readily predicted by AlphaFold (*SI Appendix*, Fig. S9*E*). Multiple groups of translation-targeting RNase TA toxins have been characterized experimentally, and display considerable diversity, even within closely related groups; for example, different VapC PIN TA toxins can cleave either tRNA (55) or rRNA (56). As expected for nucleases, metabolic labeling assays indicate the majority of the validated nuclease toxins do, indeed, target protein synthesis (Fig. 7 *A* and *B* and *SI Appendix*, Figs. S17 and S18). However, unexpectedly, expression of *Thioflavicoccus mobilis* 8321 PIN_D59_ results in inhibition of incorporation of both ^35^S methionine (abrogation of translation) and ^3^H uridine (abrogation of transcription) (Fig. 7*C*). Despite this unexpected behavior, substitutions of predicted catalytic residues of the PIN_D59_ domain (D6 and E43) abrogate the toxicity. Although metabolic labeling assays with the other two PIN_D59_ toxins (from *Crenothrix polyspora* and *Candidatus Hamiltonella defensa* (formerly *Bemisia tabaci*)) do not yield clear-cut results, they are indicative of the PIN_D59_ toxin having additional toxic effects beyond specific inhibition of protein synthesis (*SI Appendix*, Fig. S18).

## Discussion

Classical TA antitoxins are modular proteins typically consisting of a DNA-binding domain involved in transcription autoregulation and a functionally independent neutralization domain that folds upon binding to the toxin in most cases (1). It has been argued that it is the combination of, on one hand, the functional decoupling between these two structural modules and, on the other hand, the disordered nature of the neutralization domains that enables antitoxin promiscuity, i.e., allows for neutralization of toxins belonging to multiple protein families by different antitoxins possessing the same DNA-binding domain (57). This model postulates that the fusion of a “linear” recognition motif that performs the neutralization, along with a DNA-binding domain is sufficient to generate a functional TA operon. Indeed, as we show here for *C. doosanense* Doc _D6_:PanA_Phd-C system, the fusion of the Phd-C region alone to a SUMO tag is sufficient to engineer a protein that efficiently counteract the Phd-C-cognate toxin in vivo (Fig. 3*D*). Given the existence of multiple TA operons with single-domain antitoxins that consist of the neutralization domain alone (58, 59), such fusion or exchange events constitute a plausible evolutionary pathway that could generate the complex TA permutations observed in TAs (Fig. 1).

**Fig. 8.**
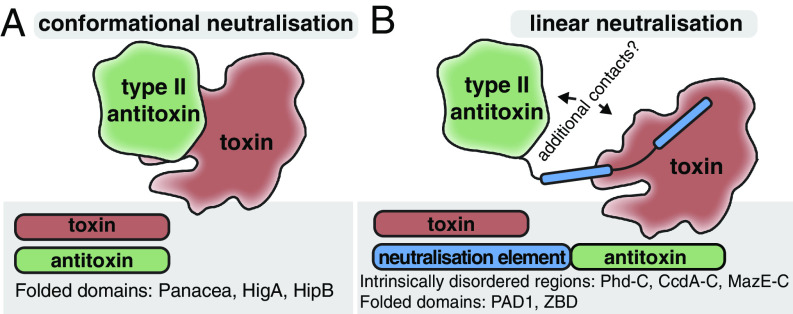
Conformational and linear neutralization are two primary modes of toxin inactivation by hyperpromiscuous antitoxins in type II TA systems. (*A*) Conformational neutralization involves the direct recognition of toxins by a three-dimensional epitope of the antitoxin that forms part of the antitoxin fold and cannot be grafted to a different scaffold. Panacea and HTH domains represent members of this class. (*B*) Linear neutralization displayed by modular antitoxins characterized by the neutralization of toxins by exchangeable elements that can be intrinsically disordered or fully folded domains. Neutralizing elements are linearly attached to antitoxin domains at the N- or C-terminal ends. This is seen in the case of Panacea linked to Phd-C, ZBD or PAD1, which connects Panacea with the neutralization of Doc, MqsR and toxSAS toxins.

Importantly, the NetFlax network reveals the existence of a type of hyperpromiscuous antitoxin that defies this commonly accepted neutralization paradigm. Such antitoxins are epitomized by Panacea and HTH domains (14, 42, 60–62) that possess within their structural fold an intrinsic capacity to specifically recognize and neutralize diverse toxins via three-dimensional epitopes (Figs. 3*C* and 8). Furthermore, these antitoxin domains can also acquire linear epitopes consisting of intrinsically disordered regions or well-folded domains, to neutralize toxins in the “classical” manner (Figs. 3 *D*–*G* and 8). In addition to this hyperpromiscuity, antitoxins have a capacity for moonlighting in other ways: PAD1-like domains are involved in both toxin neutralization and phage detection (22), while HigA antitoxins from toxin–antitoxin–chaperone (TAC) operons engage both the toxin and the dedicated regulator chaperone (63). The ability of antitoxins to readily remodel their domain combinations and evolve multifunctional moonlighting abilities of the constituent domains may be a core feature of TA roles in innate immunity involved in phage defense.

In the case of PanAs that function via linear neutralization without the direct involvement of the globular Panacea domain, the question is what then is the role of the Panacea domain. We hypothesize that its function is primarily sensory, reacting to a trigger and activating the toxin through an allosteric mechanism involving the neutralization region. This activation may not even require dissociation of the PanAT complex; the fused toxSAS TA CapRel shows that antitoxins do not have to dissociate in order to activate the toxin (22). Further investigations are needed to determine what is the functional role of the Panacea domain.

Our structural prediction has allowed the clustering of predicted toxins and antitoxins into identifiable fold classes. This tendency of TA systems to reuse folds has previously been noted (42). However, conservation of fold may not mean conservation of function: Proteins can be divergent at the sequence level—even to the point of carrying out different biochemistry, despite being based on the same structural fold. For example, the Fic/Doc family of toxins contains members that can both NMPylate or phosphorylate their protein targets (45, 64). Similarly, RelE/ParE family members with the same fold can either cleave RNA or inhibit DNA gyrase (65). Recently, we found that homologous toxSAS TAs can inhibit bacterial growth by either producing the toxic alarmone (pp)pApp or pyrophosphorylating the 3′ CCA end of tRNA (9, 66). Our *T. mobilis* PIN_D59_ domain toxin that seems to inhibit transcription as well as translation may indicate another example of a divergent function on a similar fold.

The biological function of TAs has remained a contentious subject for decades (1, 2, 67), but increasingly a role for these systems in phage defense is being discovered (15–21, 68). Here, we show that this is also reflected in their combinatorial evolutionary relationships: The core network of TAs is connected to domains in other phage defense systems. Indeed, since it is common for multigene defense systems to contain a toxic effector, it is unclear whether there is any meaningful distinction between the two kinds of system. This raises the question of whether classical “addiction module” TAs on mobile elements may actually have a role in defense against phages or competition with other mobile elements, with addiction effects being a secondary consequence.

NetFlax is a broad stroke approach, which has its limitations and caveats that we acknowledge. First, it is limited to a representative set of proteomes, which means we are missing a substantial amount of diversity. Some known type II TAs such as DarTG (69), HEPN-MNT (70), HicBA (71), and HipBA (72) escaped our prediction. Second, NetFlax only addresses conserved two-gene proteinaceous systems and therefore cannot in this incarnation predict multigene toxin–containing systems. It is also at risk of predicting false positives due to spurious domain associations. Nevertheless, despite these caveats, the network is a starting point for exploring multiple avenues including the TA systems we have characterized, and more fine-grained prediction can be achieved through a subsequent focus on specific lineages.

## Materials and Methods

### NetFlax Strategy.

TA pairs were predicted with the Python script NetFlax, which is a modification of our FlaGs program. Briefly, NetFlax works round by round; and each round follows three steps: i) scanning proteomes using an HMM profile of toxin or antitoxin to identify its protein homologues, ii) prediction of conserved TA-like arrangements and identification of homologous clusters of toxins or antitoxins and iii) cross-checking if the predicted clusters are novel (not encountered in any previous round) and, if so, make their HMM profiles, to be used in the next round of scanning.

NetFlax uses a local database of 24,474 predicted proteomes downloaded from the NCBI RefSeq FTP server (73). This includes one representative proteome per species of bacteria and archaea, along with all 10,449 available virus genomes (not limited to representatives). See *SI Appendix*, *SI Text* and *SI Appendix*, Fig. S1 for illustrations of how the algorithm works.

### Protein sequence and structure analysis.

Protein domains and other functional predictions were carried out with searching the toxin–antitoxin database (TADB) (31), DefenceFinder (25), NCBI conserved domain database (CDD) (74) and HHPred (75) (with NCBI-CDD, Pfam-A and PDB as target databases). Protein–protein complex structures were predicted with FoldDock (27). Structures were clustered with FoldSeek v. 5 (76) and with resulting networks visualized with Cytoscape v. 3.5.0 (77). Structural alignment was carried out with mTM-Align v. 20220104 (78). Transmembrane prediction was carried out with DeepTMHMM (79). More detailed methods are described in *SI Appendix*, *SI Text* document. All structures and scores are available at https://github.com/GCA-VH-lab/NetFlax_data (84).

### Experimental Methods.

Detailed experimental procedures are provided *SI Appendix*, *SI Text* document, with a summary below.

#### Plasmid construction.

All bacterial strains, plasmids, and primers used in the study are listed in Dataset S2. Toxin ORFs were cloned into an arabinose-inducible pBAD33 vector (80) either with or without Shine–Dalgarno sequence as required, for toxicity assays. For neutralization assays, antitoxins were expressed from an IPTG-inducible pMG25 vector (81). Mutations and truncations were introduced as described earlier.

#### Toxicity neutralization assays.

Toxicity-neutralization assays were performed on Lysogeny broth (LB) agar plates. First, the pBAD33 vector with the toxin ORF was transformed into competent cells of *E. coli* BW25113 strain with a pMG25 empty vector. A single colony with two plasmids was grown in liquid LB medium supplemented with 100 μg/mL ampicillin (Sigma-Aldrich) and 20 μg/mL chloramphenicol (AppliChem) as well as 0.2% glucose. Serial 10-fold dilutions were spotted (5 μL per spot) onto LB plates containing ampicillin and chloramphenicol under repressive (0.2% glucose) or induction conditions (0.2% arabinose combined with 0.05 or 0.5 mM IPTG). Plates were scored after an overnight incubation at 37 °C. After confirming toxin toxicity, another set of competent cells was produced with the cognate antitoxin in a pMG25 vector, and the spot test was repeated.

#### Metabolic labeling.

Metabolic labeling experiments using *E. coli* BW25113 strains cotransformed with pBAD33 derivatives as well as the empty pMG25 vector were performed as described earlier (14).

#### Fluorescence microscopy.

Fluorescence microscopy experiments with SYTOX Green (82) and DiSC_3_(5) (83) were performed as described previously (14).

#### Phage immunity assays.

Phage immunity assays using BASEL coliphages and *E. coli* BW25113 strains transformed with pJD1423-based plasmids (pBR322 derivative) expressing TA systems under the control of P_tet_ promoter were performed as per Maffei *et al.* (50).

## Supplementary Material

Appendix 01 (PDF)

Dataset S01 (XLSX)

Dataset S02 (XLSX)

Movie S1. ***G. mesophilus* toxFtsL_D9_ triggers cell elongation and FtsZ delocalization in *E. coli***. Legend: Time lapse microscopy of *E. coli* BW25113 cells expressing YFP-FtsZ and toxFtsL_D9_. The cells grown in MOPS/glycerol medium in the presence and absence of the inducer arabinose were imaged for 220 min at 5 min intervals. The movie frame rate is 5 fps. Scale bar, 3 μm.

## Data Availability

Code and structures data have been deposited in Github (https://github.com/GCA-VH-lab/NetFlax_data) (84). All study data are included in the article and/or supporting information.
